# Perception of facial profile attractiveness of a brown subject displaying different degrees of lip projection or retrusion, in the city of Salvador/Bahia

**DOI:** 10.1590/2177-6709.23.2.062-067.oar

**Published:** 2018

**Authors:** Elizabete Nobre Carneiro, Matheus Melo Pithon, André Wilson Machado, Emanuel Braga

**Affiliations:** 1 ​​Private practice (Salvador/BA, Brazil).; 2 Universidade Estadual do Sudoeste da Bahia, Curso de Odontologia (Vitória da Conquista/BA, Brazil).; 3 Universidade Federal da Bahia, Faculdade de Odontologia (Salvador/BA, Brazil).

**Keywords:** Face, Profile, Protrusion, Skin color, Orthodontics.

## Abstract

**Introduction:**

The attractiveness and facial aesthetics are commonly defined by the media in modern society and the number of patients seeking for changes in the facial appearance is progressively increasing. Augmented face convexity is believed to be non aesthetic and among the treatments available for its correction, the extraction of premolars followed by anterior teeth retraction produces a significant effect. However, it is questionable whether the procedure is reasonable in brown and black patients, since dental protrusion is considered a common morphological feature in these groups.

**Methods:**

A photograph of a brown female subject was manipulated with image-editing program to generate a set of images with different degrees of labial retraction/protrusion. Two hundred individuals, randomly selected, were asked to rate each photograph and select which one showed the best aesthetic appearance. The survey was carried out in Salvador / Bahia (Brazil), which is a city with strong African slavery background and high proportion of brown and black population.

**Results:**

Regardless of color, sex or income, the interviewees chose primarily the straight facial profile and secondarily the slightly concave or convex as more pleasant for a brown female individual (*p*< 0.05). The moderate and extreme convexity had the lowest scores (*p*< 0.05).

**Conclusions:**

It is suggested that among the accessed population, straight and slightly convex or concave profiles were preferred for a brown subject and moderate or excessive facial protrusion were not well accepted. It is expected that these data can guide orthodontists about the need for extraction to reduce dental protrusion in brown patients.

## RESUMO

**Introdução:**

a atratividade e a estética facial são comumente definidas pela mídia, na sociedade moderna, e o número de pacientes buscando mudar seu aspecto facial tem aumentado progressivamente. Acredita-se que a convexidade facial aumentada seja considerada não estética. Entre os tratamentos disponíveis para sua correção, figura, com destaque, a extração de pré-molares seguida da retração dos dentes anteriores. No entanto, é questionável se esse procedimento estaria indicado para indivíduos negros e pardos, visto que a protrusão dentária é considerada um achado morfológico comum nesses grupos.

**Métodos:**

a fotografia de um indivíduo pardo do sexo feminino foi manipulada em programa de edição de imagens, de forma a gerar um grupo de imagens com diferentes graus de protrusão ou retrusão labial. Duzentos indivíduos, selecionados aleatoriamente, avaliaram e foram solicitados a escolher a fotografia que melhor se apresentava esteticamente. Essa pesquisa foi executada em Salvador / Bahia (Brasil), que é uma cidade com marcante histórico de escravidão africana, apresentando elevada proporção de negros e pardos.

**Resultados:**

independentemente da cor, sexo ou renda, os entrevistados escolheram como mais estético, para um indivíduo pardo, o perfil reto, seguido do perfil levemente côncavo ou convexo (*p*< 0,05).

**Conclusões:**

dentro da amostra entrevistada, o perfil reto foi considerado mais estético para um indivíduo pardo, seguido do perfil levemente côncavo ou convexo. Os perfis moderadamente e excessivamente convexos foram considerados menos atraentes. Espera-se que esses achados possam guiar os ortodontistas no que diz respeito à necessidade de extrações dentárias, com intuito de reduzir a protrusão dentária/labial em pacientes pardos.

## INTRODUCTION

Facial aesthetics can play an important role in interpersonal relationships, social inclusion and self-esteem. It is also believed that facial appearance can determine relevant psychological aspects of some individuals. In this context, it is increasing the number of patients seeking for therapies that can modify facial appearance and the orthodontic treatment has clearly become an indispensable approach.^1^ Well established features may not represent the real need of different countries or regions with distinct cultural and historical backgrounds.^2^ ^,^ ^3^ Based on the exposed, researches addressed to reveal what is considered beauty or aesthetically acceptable are anticipated for different regions and it is believed that the results can somehow guide the treatment planning. 

Regarding facial profile, jaw position and incisors inclination play an important role on face display and offer relevant information for treatment planning.^1^ ^,^ ^4^ In the group of brown and black individuals, bimaxillary protrusion and incisors buccal inclination are common features when compared to Caucasian subjects.^10^ Among the treatments available for reduction of face convexity, extraction of premolars followed by anterior teeth retraction produces a significant effect;^4^ however, it is questionable whether the procedure is reasonable in brown and black patients, since dental protrusion may be considered a normal morphological feature in these groups. Moreover, available cephalometric analysis may not be completely suitable for employment in brown and black subject’s treatment planning, since the patterns are usually brought from white Caucasian samples^5^. Orthodontic planning should take great consideration of these features specially in regions with important racial diversity.^6^ ^,^ ^7^

The demand from brown and black individuals aiming to reduce face convexity is still speculation and object of investigation. Previous researchers have identified the protrusive profile as the most attractive for a black South African sample, without differences between male and female responses.^8^ Accessing a sample of black South Africans, another study has found that, if needed, interviewees would be willing to be orthodontically treated to achieve the most attractive profile displayed in the study.^9^ An study conducted with black Americans demonstrated that the slightly convex profile was considered the most attractive facial appearance, regardless of the interviewees skin color (black or white) or occupation (layperson , orthodontist or general dentists).^10^

Brazil is a continental size country with great racial diversity, making clear the need for better understanding about facial attractiveness in this society. Regarding the Brazilian population, Pithon et al^11^ evaluating photographs and silhouettes have show that the slightly concave profile was considered the most attractive for a brown subject, and male and female have not presented divergent responses. Melo et al^12^ evaluating Brazilian black individuals facial appearance demonstrated that the displayed profiles were considered aesthetically acceptable and identified the disharmony of chin and nose as the relevant factor for profile unpleasantness. 

Salvador is a city with strong African slavery background and exhibits one of the highest proportions of brown and black population among the Brazilian cities. In this context, the null hypothesis is that bimaxillary protrusion is well accepted among this population, since it is expected to be a common feature. The present study aims at accessing the facial profile preference for a brown subject in the city of Salvador and by this mean bring out insights for orthodontic planning in regions with great racial diversity. 

## MATERIAL AND METHODS

This descriptive study was carried out in the city of Salvador, Bahia state, Brazil. Interviewees were approached on the streets or in public places such as bus terminal and parks or in supermarkets and shopping centers. The research was explained in detail and the ones who agreed in participating in the study signed an informed consent term. Researchers contact was given and anyone could withdraw the consent and quit the participation at any time. The research was approved by the Research Ethics Committee, Faculty of Dentistry, Federal University of Bahia (#35868614.7.0000.5024).

**Figure 1:**
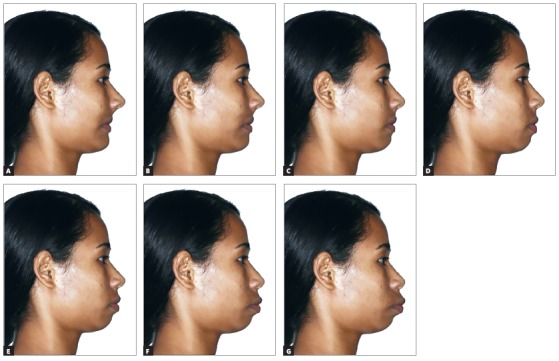
Degrees of lip retrusion or protrusion. A) - 4.0 mm; B) - 2.0 mm; C) 0 mm; D) + 2.0 mm; E) + 4.0 mm; F) + 6.0 mm; G) + 8.0 mm

In order to conduct this study, the image of the profile of a brown woman was used (Fig 1). The patient’s consent to use the photograph and teleradiograph was obtained by means of signature of a term of free and informed consent to undergo orthodontic treatment, stated on the respective patients’ clinical record charts. The pretreatment photographic image was manipulated using the program Photoshop CS6, Version 13.0 (San Jose, Calif, USA) to produce different lip positions with alterations of 2 mm. The study published by Pithon et al^11^ served as a basis for this research. Intervals of 2 mm were determined so that evaluators would be capable of detecting differences between dental esthetic alterations. 

All the alterations were limited to the anteroposterior dimension, and there were no alterations in the vertical dimension. The structures manipulated were the soft tissues between the subnasal points and mentolabial sulcus. The initial photographic image was changed in relation to Ricketts’ line E, producing positions of -4mm (Fig 1A), -2mm (Fig 1B), 0 (Fig 1C), +2mm (Fig 1D, original), +4mm (Fig 1E) , +6mm (Fig 1F), and +8 mm (Fig 1G) in the lip prominence. Thus, the original and six modified photos were obtained. The seven photographs were placed in a set in a single slide, so that there were seven photographs per slide (10 x6 cm), which were randomly numbered and printed in proper album, using photographic paper. Images were thus presented to the evaluators. The present work is a complementary part of the study published previously by Pithon et al.^11^ The sample size was calculated using ANOVA for repeated measures. The power of the test was 80% (β = 0.20) and the error was 5% (α = 0.05). Calculation determined the minimum of 82 individuals. Considering the possibility of non parametric statistics (Friedman), 15% of addition was recommended, according to Lehmann.^13^ Attempting to enhance the power of the test, 200 individuals were invited. Sample size was performed using the program G Power Version 3.1.9.2 A semi-structured questionnaire was designed for the presented study comprising the main question “From the profiles below, which one do you consider the most pleasant?”, and a demographic survey inquiring gender, age, income and skin color. Evaluators under 18 years of age were not selected for the study. Evaluators who did not want to fill any of the questions were excluded. Dentists and dental students were also not eligible. In the present study, skin color was accessed by self-definition, according to the Brazilian Government guidelines.^14^

### Statistical analysis

The responses of the interviewees regarding the most pleasant profile were expressed as frequencies relative to the respective confidence intervals (CI) set at 95%. The association of the responses given by the evaluators was compared according to gender, skin color, income and age, by means of the Fisher exact test. For all statistical analyses, a level of significance of 5% (*p*< 0.05) was adopted. The data were analyzed with the statistical software IBM SPSS Statistics for Windows (IBM SPSS. 21.0, 2012, Armonk, NY: IBM Corp.). Method error was tested prior to the beginning of the research and showed good reproducibility.

## RESULTS

The demographic data obtained from the interviewees is shown in Table 1. The sample was well balanced regarding gender. A great number of participants belonged to the <40 years of age range and received three or less Brazilian minimum wage as income. Regarding the skin color, the sample consisted of 39% of brown, 36% black and 25% white. 

**Table 1:** Sample demographic characteristics.

SAMPLE	n	%
GENDER		
male	109	54.5
female	91	45.5
AGE RANGE (years)		
≤ 20	16	8.0
21 to 30	44	22.0
31 to 40	54	27.0
> 40	86	43.0
SKIN COLOR		
black	72	36.0
white	50	25.0
brown	78	39.0
INCOME (Brazilian Minimum Wage)		
≤ 3	135	67.5
> 3 to 10	42	21.0
> 10	23	11.5

**Figure 2:**
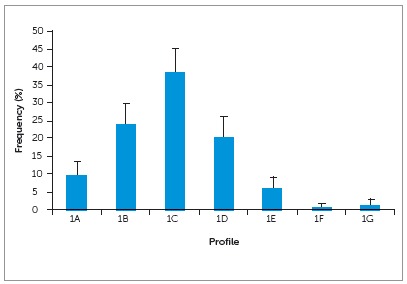
Interviewees preference regarding the most pleasant profile. Columns indicate relative frequencies (%) and error bars indicates the confidence intervals (CI 95%).

The distribution of the answers regarding the most pleasant profile is presented in Figure 2. Profile 1C (0 mm) was significantly more attractive (38.5%; CI95%: 31.8% - 45.2%), followed by profile 1B (-2mm) (24.0%; CI95%: 18.1% - 29.9%) and 1D (+2mm) (20.5%; CI95%: 14.9% - 26.1%). The profile 1F (+4mm) (0.5%; CI95%: 0.0% - 1.92%) and 1G (+6mm) (1.0%; CI95%: 0.0% - 3.0%) did not present difference to each other and were considered the less attractive. 

The answer regarding the attractiveness of each of the profiles could not be associated to gender (*p*= 0.329), skin color (*p*= 0.199) or income (*p*= 0.199) of the participants. However, positive correlation could be found regarding age (Table 2). The preferred image (profile 1C) had better acceptance among the group belonging to 31 to 40 years old range, followed by younger groups (≤20 and 21 to 30 years), respectively. Profile 1B was more accepted among the group of ≤ 20 years and > 40. Profile 1D present less acceptance among the participants belonging to 31 to 40 years age range. Profiles 1E, 1F and 1G did not vary significantly throughout the age groups. 

**Table 2:** Association between interviewees preference regarding the most pleasant profile and age range.

PROFILE	AGE RANGE				p*
	≤ 20 years	21 to 30 years	31 to 40 years	> 40 years	
1	0 (0.0%)	5 (11.4%)	3 ( 5.6%)	11 (12.8%)	0.005
2	6 (37.5%)	8 (18.2%)	9 (16.7%)	25 (29.1%)	
3	7 (43.8%)	18 (40.9%)	34 (63.0%)	18 (20.9%)	
4	3 (18.8%)	11 (25.0%)	3 (5.6%)	24 (27.9%)	
5	0 (0.0%)	2 (4.5%)	3 (5.6%)	7 (8.1%)	
6	0 (0.0%)	0 (0.0%)	1 (1.9%)	0 (0.0%)	
7	0 (0.0%)	0 (0.0%)	1 (1.9%)	1 (1.9%)	

## DISCUSSION

Based on the revised literature, it is evident that the orthodontic planning must take in consideration the racial and morphological peculiarities of each patient. Brown and brown individuals commonly display a more protrusive profile and the available cephalometric analysis should be used with care when planning for these group of patients.^8^ ^,^ ^9^

Bimaxillary protrusion is considered a common feature for the black individuals^4^ ^,^ ^5^ and the real need for facial convexity reduction in this group is still object of investigation. In this regard, the current study was carried out to clarify the perception of the population of the city of Salvador about the facial profile attractiveness for a brown subject. This city is particularly interesting for the execution of the study, since it has historically experienced strong African slavery background and exhibits one of the highest proportions of brown and black population among the Brazilian cities. The most updated screening revealed that 79,5% of the population self-declared as brown and black skin (51,7% brown and 27,8% black).^14^ In this context, it was speculated that bimaxillary protrusion could be well accepted among this population, since it is expected to be a common feature. 

The null hypothesis was rejected. Interestingly, the findings of the present study have shown that the profile 1C (0mm) was significantly (*p*< 0.05) elected as the most pleasant, followed by profile 1B (-2.0mm) and 1D (+2mm), without difference among each other. Profiles 1F (+4.0mm) and 1G (+6.0mm) did not present any difference among each other and were considered the less attractive. Since the original photo presented a slight protrusion, it can be inferred that the participants elected the straight profile as the most pleasant image, followed by the slight concave and slight convex. 

The results are in consonance with Pithon et al.^11^ that have also shown the preference for straight profiles in Brazil. The authors, however, have accessed a different sample composed by dental students. Conversely, the present findings diverge from studies performed in Brazil by Okuyama and Martins,^15^ which have shown that laypeople, orthodontists and visual artists preferred more protruded lips for black individuals when compared to white or yellow. The present findings also diverge from studies performed in other countries^8^ ^,^ ^9^ ^,^ ^10^ that have clearly demonstrated a preference for slight and moderate convex profiles for black individuals. These findings highlight the importance of regional background regarding facial aesthetics; however, many years have passed since the execution of those studies and aesthetics perception may change drastically within a short period of time. It is believed that such information should be updated more often. In addition, accessing different sample configuration from distinct countries may be an explanation for the divergent results.

Regarding the demographic aspects tested (Table 1), the answers could not be correlated with gender, income and skin color of the participants. Positive but not clearly specific correlation could be found regarding age. The preferred profile had better acceptance among the group belonging to 31 to 40 years old range. In addition, the proportion of white, black and brown participants followed similarly the proportion found in the city of Salvador, ensuring the representativeness of the sample. 

Previous studies have emphasized the need for individualization of the treatment planning for brown and black patients in Brazil.^16^ ^,^ ^17^ Other study also performed in Brazil have highlighted that the aesthetic perception of the profile vary depending on the tested region.^18^ Certainly, the present study has limitations such as sample configuration displaying regional preference. More studies are anticipated to understand this matter.

In summary, face protrusion can be considered a common morphological feature of brown and black individuals and it seems to exist a consensus that the orthodontic planning should be individualized following proper parameters for the different racial groups^16^ ^,^ ^17^. The findings of the present study have revealed, in the accessed sample, a preference for the straight profile for a brown female subject. It is thus suggested that there is a clear need for patient enrollment in the planning decisions. 

## CONCLUSIONS

It is suggested that among the accessed population, straight and slightly convex or concave profiles were preferred for a brown subject and moderate or excessive facial protrusion received the lowest scores regarding facial profile attractiveness. Regarding the demographic aspects, the results could not be correlated with gender, income and skin color of the participants.
